# The Logic of Surveillance Guidelines: An Analysis of Vaccine Adverse Event Reports from an Ontological Perspective

**DOI:** 10.1371/journal.pone.0092632

**Published:** 2014-03-25

**Authors:** Mélanie Courtot, Ryan R. Brinkman, Alan Ruttenberg

**Affiliations:** 1 BC Cancer Agency, Vancouver, British Columbia, Canada; 2 Department of Medical Genetics, University of British Columbia, Vancouver, British Columbia, Canada; 3 School of Dental Medicine, University at Buffalo, New York, United States of America; University of Missouri Kansas CIty School of Medicine, United States of America

## Abstract

**Background:**

When increased rates of adverse events following immunization are detected, regulatory action can be taken by public health agencies. However to be interpreted reports of adverse events must be encoded in a consistent way. Regulatory agencies rely on guidelines to help determine the diagnosis of the adverse events. Manual application of these guidelines is expensive, time consuming, and open to logical errors. Representing these guidelines in a format amenable to automated processing can make this process more efficient.

**Methods and Findings:**

Using the Brighton anaphylaxis case definition, we show that existing clinical guidelines used as standards in pharmacovigilance can be logically encoded using a formal representation such as the Adverse Event Reporting Ontology we developed. We validated the classification of vaccine adverse event reports using the ontology against existing rule-based systems and a manually curated subset of the Vaccine Adverse Event Reporting System. However, we encountered a number of critical issues in the formulation and application of the clinical guidelines. We report these issues and the steps being taken to address them in current surveillance systems, and in the terminological standards in use.

**Conclusions:**

By standardizing and improving the reporting process, we were able to automate diagnosis confirmation. By allowing medical experts to prioritize reports such a system can accelerate the identification of adverse reactions to vaccines and the response of regulatory agencies. This approach of combining ontology and semantic technologies can be used to improve other areas of vaccine adverse event reports analysis and should inform both the design of clinical guidelines and how they are used in the future.

**Availability:**

Sufficient material to reproduce our results is available, including documentation, ontology, code and datasets, at http://purl.obolibrary.org/obo/aero.

## Introduction

The importance of pharmacovigilance as a tool for global health policies has been well described [1], even more so in vaccine risk communication, which has been shown [2] to have a direct impact in decisions to immunize in the general public, and is a probable underlying cause in the recent resurgence of vaccine-preventable diseases such as pertussis [3] or even the current (September 2013) measles outbreaks [4], [5]. Efficient analysis of adverse event reports is a time-consuming task, requiring qualified medical personnel. For example, a team of 12 medical officers worked for over three months to review 6,000 post-H1N1 immunization reports for positive cases, only a fraction of the total number of reports received [6]. Automated approaches that address this bottleneck have the potential to reduce this significant burden of costly manual review and facilitate timely follow-up of positive reports.

## Background

### Surveillance of Adverse Events Related to Immunization

Reports of Adverse Events Following Immunization (AEFIs) are important elements in the assessment of safety of vaccines and play a major role in public health policy. For immunization campaigns to be effective the general population needs to be adequately informed so that they maintain confidence in and trust individuals responsible for managing vaccination efficiency and safety [2]. Prior to licensing and market approval vaccines are tested, through randomized clinical trials, for efficacy and safety. However, the focus of those trials is efficacy, particularly in the case of widespread, easily transmissible infections such as influenza where it is hard to fully assess safety due to the limited number of subjects. Effects in the larger population and in specific subpopulations such as children, pregnant women, the elderly and people likely to experience an adverse event such as those with history of autoimmune disorders etc. can only be studied post-licensing. It has been shown that this leads to underestimation of the occurrence of adverse events once the vaccine is licensed [7], [8]. Similarly, chronic effects, or effects of concomitant administration of other drugs, become evident only after several years of surveillance. As a consequence, there is a need to encourage long-term, widespread post-licensing surveillance. Generally, spontaneous reporting systems are used to monitor for adverse effects in the general population [9]. Analysis of events in large collections of AEFI reports aims to identify signals highlighting differences in frequency of events after administration of a certain vaccine (e.g., a seasonal influenza vaccine), or in certain populations (e.g., children under the age of 2). When such signals are detected health authorities use that information to prompt investigation of a risk of potential safety issues. Depending on their findings, health officials can make choices such as withdrawing the vaccine from general use or mandating further clinical studies.

### Brighton Collaboration

To allow for comparability of data, it is desirable that a global standard for case definitions and guidelines be used for AEFI reporting [10]. The Brighton Collaboration [11], a global network of experts who aim to provide high quality vaccine safety information, has done extensive work towards this end [12]. However, and despite their completeness, the textual, article-like format of the Brighton case definitions makes it both problematic for clinicians to confirm that they see the relevant symptoms when making the adverse event diagnosis and difficult to automate [13].

#### Automatic Brighton Classification (ABC) tool

The ABC tool [14] is the only automated classification system that allows its users to work with the Brighton case definitions. Given a set of symptoms and a tentative diagnosis, one can confirm the level of diagnostic certainty of an AEFI. Or, given a set of symptoms, the tool can compare them to all Brighton case definitions and report putative diagnoses and their probabilities. Three limitations warrant development of an ontology for the purpose of either revising the ABC tool, or to form the basis for a new tool.

1. Signs and symptoms are not defined within the Brighton tool making it hard to know if individual findings are the same type as those described in the case definitions.
2. The tool is embedded within the Brighton portal with access only by permission and after legal agreement between the requesting party and the Brighton Collaboration. Therefore the ABC tool cannot be incorporated into third-party systems and only remote access use of the tool is available, limiting the widespread adoption of the guidelines.
3. The rules of classification are part of the software implementation of the tool rather than being able to be declared independently. Because a computer programmer is required to make changes, it is harder to maintain and be extended. As new case definitions are developed by members of the Brighton community, if the tool implementation were independent from the case definitions it would be cheaper and easier to keep the tool accurate and up-to-date.

### Ontology

The term ‘ontology’ is used in a number of different ways in technical communities. In our case, building an ontology means addressing three essential roles [15]:

1. An ontology in a given domain is a collection of representations of the important types of things in that domain, with an understanding that instance representations any of these types should be considered proxy for things in the world. The truth of assertions made on the representations is judged by the facts about that which the representations serve as proxies.
2. An ontology is an active computational artifact. The assertions that are made are in a subset of first order logic which can be checked for consistency and from which logical consequences can be computed. We aim to take advantage of this by asserting as many axioms as feasible. As a way of improving quality, these axioms maximize the opportunity for consistency checks to turn up errors. We classify each case report when the specifics of the case satisfy a guideline for the purpose of diagnosis and screening. For example we can ask for all the cases of adverse effects that affect a specified anatomical system such as ‘skin rash located at some dermatological-mucosal system’.
3. An ontology facilitates scholarly and technical communication. In our case the this includes a) working in a large community of ontology developers who split the labor and use each other’s work [16], and who together work out principles that encourage quality through careful analysis, b) mediating communication between clinicians and technical specialists when we follow our practice of having literate documentation about the types in the ontology, so that clinicians and co-workers can have reasonably expectations of what our data means, and c) serving as an artifact that is part of the package distributed so that other researchers can reproduce our results.

## Methods

### AERO Ontology

The Adverse Events Reporting Ontology (AERO) leverages the Brighton guidelines by embedding them in the Semantic Web framework [17]. Within that computable framework we can automate the classification of reports of adverse events and so improve the efficiency of discovering potential risks. When developing the AERO, care was taken to reuse, when possible, work done in the context of other efforts. Reusing terms from other resources allowed us to rely on knowledge of domain experts who curated them and to dedicate more time to defining new terms [18]. AERO reuses terms from the Open Biological and Biomedical Ontologies (OBO) Foundry [16] suite of ontologies that cover an ever-increasing range of entities related to biology and clinical practice.

#### *Assessment pipeline*

**Figure 1:**
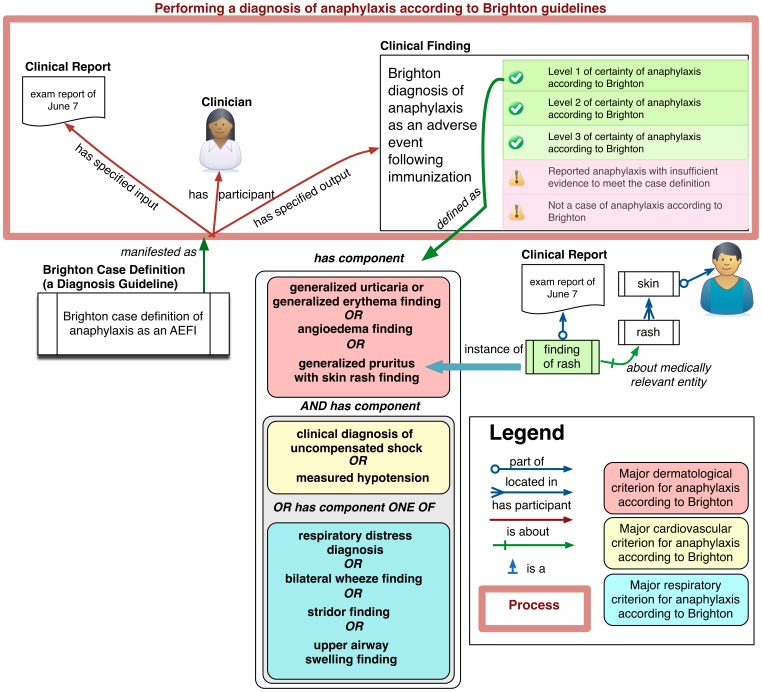
The elements of an assessment of anaphylaxis according to Brighton as implemented in AERO. Performing a diagnosis involves assessing a number of criteria each (e.g. lower middle box) implemented as a class expression that classifies a set of findings. The diagnosis of Level 1 of certainty of anaphylaxis is made by the clinician if the written criteria apply, and by our OWL implementation if the class expression subsumes the set of findings shown in illustration as a *Clinical Report*.

**Figure 2:**
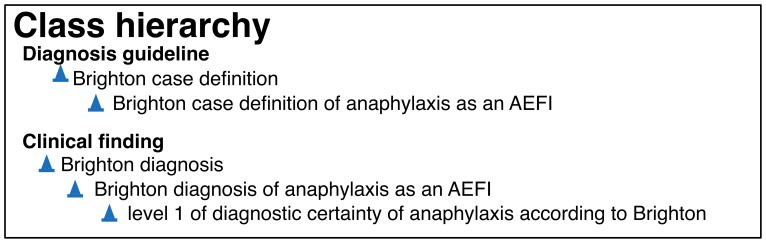
Class hierarchy excerpt in the AERO. Every ‘level 1 of diagnostic certainty of anaphylaxis according to Brighton’ is a ‘Brighton diagnosis of anaphylaxis as an AEFI’, which is in turn is a ‘Brighton diagnosis’, itself a ‘clinical finding’. Every ‘Brighton case definition of anaphylaxis as an AEFI’ is a ‘Brighton case definition’, which in turn is a ‘diagnosis guideline’.

Figure 1 shows how the various entities are related in AERO to form a diagnosis pipeline for the assessment of anaphylaxis according to the Brighton guideline. The patient examination by the physician results in a set of clinical findings that are part of a report, upper left. The report findings are input to a process of diagnosis which uses the case definition. We say the case definition is *concretized as* the plan to use the guidelines in a process of diagnosis, and that this process *realizes the plan* (in figure as *manifests as*). The case definition includes different criteria concerning the findings, each of which, when satisfied, yields some assessment of the certainty of anaphylaxis being present. For example, the lower middle stack represents the criteria for diagnosing a *level one of certainty according to the Brighton anaphylaxis guideline*. When findings in the report together satisfy these criteria, the output of the diagnostic process is determination of the level 1 of diagnostic certainty of anaphylaxis according to Brighton. Figure 2 gives two extracts from the class hierarchy related to terms in the figure. It reads: Every ‘level 1 of diagnostic certainty of anaphylaxis according to Brighton’ is a ‘Brighton diagnosis of anaphylaxis as an AEFI’, which is in turn is a ‘Brighton diagnosis’, itself a ‘clinical finding’. Every ‘Brighton case definition of anaphylaxis as an AEFI’ is a ‘Brighton case definition’, which in turn is a ‘diagnosis guideline’.

### VAERS Dataset

The Vaccine Adverse Event Reporting System (VAERS) [9] is a post-market passive surveillance system, under joint authority from the Centre for Disease Control (CDC) and the US Food and Drug Administration (FDA). It provides self-reporting tools for individuals and health practitioners, and its datasets are publicly available. VAERS reports are semi-structured. A free text field contains the report notes, and another field contains a list of Medical Dictionary of Regulatory Activities (MedDRA) terms that correspond to the report.

The dataset described in [6], was obtained through a series of Freedom Of Information Act (FOIA) requests. It consists of 6034 reports received between the end of 2009 through early 2010, all following H1N1 immunization after the FDA was alerted of a possible anaphylaxis safety signal by the Canadian Health Agency. However data surrounding the 100 confirmed anaphylaxis cases in the original report were unobtainable as they were deemed lost. All reports in this set were evaluated by specialists and so provide a gold standard for comparison. A series of FOIA requests were also used to obtain the dataset describing classification results on the same dataset using the ABC tool, the Standardized MedDRA Queries (SMQs) as well as a custom information retrieval method [19]. However details of the original analysis approach necessary for reproducing the original results were not made available and we could only hypothesize the cause of results we obtained that were not in concordance with the original publication. To demonstrate that the AERO can be used to effectively encode a logical formalization of the Brighton guidelines, we compared the output of classification using the ABC tool with the results of the classification using the ontology.

### Data Loading and Processing

To streamline the analysis process, we used Python to perform the following steps, semi-automatically:

1. Load the VAERS reports into MySQL. The VAERS data was provided as a set of Excel spreadsheets, and MedDRA is distributed as ASCII files and corresponding database schema. Both were loaded into a relational database for easier processing.
2. Apply the mapping (see [19], Electronic Supplementary Material, Appendix 3) from the existing MedDRA annotations to the Brighton terms. Each MedDRA ID was mapped to the corresponding AERO ID, and a mapping table was created in the database. As working with the complete dataset in OWL was neither efficient nor necessary we partitioned the data into smaller files as follows.
3. Export the dataset into a series of Resource Description Framework (RDF) files and perform pre-processing. For each report (i.e., each VAERS ID), we collected all information in that report for RDF serialization. Next, MedDRA terms were mapped to assertions using AERO. Because the OWL representation required more information than was available in the reports, choices had to be made before classification could proceed, specifically (1) setting some Brighton required values to true as they cannot be encoded in the current version of MedDRA (2) add negation to reports to simulate the closed world assumption made in the reports. These steps are both further explained below. Serialization was done using the FuXI framework [20], which provides a syntax for the Web Ontology Language (OWL) [21] entities in Python that is more amenable to coding than RDF/XML.
4. Apply an OWL reasoner to classify reports. The reasoning step was performed with the HermiT reasoner [22], via the OWLAPI [23]. In series, each RDF file was loaded, the reasoner computed inferred axioms, including individual types assertions, and those axioms were recorded into another RDF file.
5. Load each of the original RDF and associated inferred axioms as well as AERO into a Sesame triplestore [24]. We found it was more user friendly to use Sesame’s interface for querying.

## Results

### Brighton Classification Results

We were able to successfully classify a subset of just over 6000 VAERS records in just over 2 h on a Mac OS X laptop with a 2.4 Ghz Intel Core i5 and 8 GB of memory. We then queried the triplestore to retrieve reports in each of the Brighton case definition categories; results are shown in Table 1. However, we ran into several issues - either with the annotation standard being used (such as MedDRA), the quality/availability of the information in the reporting systems (such as VAERS) and interpreting the guideline (such as the Brighton case definitions).

**Table 1:** Classification results.

	Positive cases					Negative cases		
	Level 1	Level 2	Level 2 updated	Level 3	Level 3 updated	Insufficientevidence	Not a case	No evidence
ABC tool	101	221	N/A	7	N/A	488	2844	2373
Ontology with negation	98	223	223	8	8	3	3078	2622
Ontology without negation	98	178	223	4	8	3	3078	2622

The first row are the results of running the ABC tool online, as described in [19]. The second row is the initial ontology-based classification, using the same rules and with the addition of the negation for information non present in the reports. The last row is the ontology-based classification without the addition of the negation. Level 1, 2 and 3 columns represent the existing Brighton classification categories. Level 2 updated and Level 3 updated represent the category as they should have been encoded based on communication with the Brighton collaboration.

First, there are critical limits to the temporality representation in MedDRA. Temporality information is needed for causality assessment: it is a necessary (though not sufficient) condition that the temporal association be consistent with the immunization. Temporality data is also needed for diagnosis determination (which is of interest for the classification) to represent dynamic disease conditions, such as onset, progression (rapid, chronic?) and relapsing. In the specific case of anaphylaxis, there are no MedDRA terms allowing encoding of ‘sudden onset’ and ‘rapid progression’ which are necessary conditions to reach any positive level in the Brighton classification of Anaphylaxis. The strict application of the Brighton guidelines to the VAERS dataset as-is would result in a value ‘don’t know’ for those criteria, and consequently classify all reports as negative (insufficient evidence/not a case). Second, there are no distinctions between unknown/missing/non applicable information in the reporting systems. In the case of ‘generalized pruritus without skin rash’, when the report does not provide any information about ‘skin rash’, it is impossible to know whether that information is unknown (the physician did not check for presence/absence of skin rash), missing (the physician did check but the information was not recorded) or was negative and therefore not included in the report (the physician checked and did not see a skin rash, but the negative finding was not included in the report).

To remedy those two major issues, and for the purpose of research, we added the condition that ‘Rapid progression’ and ‘sudden onset’ criteria are not required for diagnosis to the VAERS dataset. We also added negation of those signs or symptoms that were not positively stated on each report. For example, clinical findings of the report 369695 are defined as (shown using the Manchester syntax [25]):

Individual: 369695.

Types:

‘clinical finding’,
‘has component’ some ‘generalized erythema finding’,
‘has component’ some ‘generalized urticaria finding’,
‘has component’ some ‘difficulty breathing finding’

does not reach a Brighton level of diagnosis certainty. However, with the addition of the restrictions

not (‘has component’ some ‘bilateral wheeze finding’),
not (‘has component’ some ‘stridor finding’)

the condition for minor respiratory criteria is fulfilled (‘difficulty breathing without wheeze or stridor’) and the report is classified as Level 2 of certainty.

Third, when translating the Brighton guidelines into their logical form, we did encounter different interpretations of the same human readable content, and had extensive discussion with the Brighton collaboration to clarify part of the formalization. Upon realizing that the addition of negation to the dataset would be required (that we established was also the case in [19], though unpublished), we further enquired with the Brighton collaboration as to whether those negations were logically and clinically required or if the were added to allow human readers to distinguish between minor and major criteria. For example ‘pruritus with or without skin rash’, which is major or minor criterion respectively: ‘pruritus’ ought to be enough as minor criterion, there should be no need to require the presence of the ‘no skin rash’ (which is currently required in the ABC tool). Practically, this means that if we consider a report annotated with ‘Rapid progression of signs and symptoms’, ‘Sudden onset of signs and symptoms’, ‘Hypotension, measured’, ‘Pruritus, generalized’: with the addition of ‘Skin rash: Yes’ it classifies as expected as Level 1. With the addition of ‘Skin rash: No’ it does classify as expected as Level 2. However, with the addition of ‘Skin rash: Don’t know’ it classifies as ‘insufficient level of evidence’ - which is incorrect: even if we don’t know, there was either presence of skin rash or not, so this report should at a minimum classify as level 2 of diagnostic certainty.

Another outcome of our work is that compound terms should be represented as association of individual terms. For example, ‘capillary refill time of > 3 s without hypotension’ should be encoded as ‘Capillary refill time > 3 sec’ and not ‘Hypotension, measured’. There are currently 2 entries in the ABC tool: one can either select ‘Capillary refill time > 3 sec’ Yes and ‘Hypotension, measured’ No OR one can select ‘Capillary refill time > 3 sec, no hypotension’. While the former behaves as expected when applied to an anaphylaxis report for which ‘capillary refill time of > 3 s without hypotension’ is a cardiovascular criterion, the latter doesn’t allow for correct classification. Similarly, in the pruritus case above, ‘generalized pruritus with skin rash’ should be ‘generalized pruritus’ and ‘skin rash’. This allows differentiating between a major dermatological criterion (‘generalized pruritus with skin rash’) and the corresponding minor dermatological criterion (‘generalized pruritus’ and not ‘generalized pruritus without skin rash’). By systematically reviewing and applying this to other criteria, we were able to overcome the need for addition of negation in the dataset. We also observed that there exist different human interpretations of the same guideline, often linked to ambiguity in the textual representation of the criteria. For example, the case definition of anaphylaxis states that a level 3 of diagnostic certainty is reached when we observe:

- ≥1 minor cardiovascular OR respiratory criterion AND
- ≥1 minor criteria from each of ≥ 2 different systems/categories

This was interpreted as (1 minor cardiovascular OR respiratory criterion) AND 2 minors from systems that are neither respiratory nor cardiovascular (dermatologic, gastrointestinal, laboratory systems) and so translated in the ABC tool. However it should have been rather read as “if there is a minor cardiovascular criterion, then we need 2 other systems involved, including respiratory, dermatologic, gastrointestinal and laboratory” (and vice versa for a respiratory criterion).

Following discussion of those results with the Brighton Collaboration, we encoded and added an updated version of Brighton guidelines in the AERO, in addition to the existing ones, to reflect those changes. Based on these changes we were able to reason over our dataset, without the addition of negation, and simultaneously compare the different cases, shown in table 1 under columns ‘Level 2 updated’ and ‘Level 3 updated’ (there were no modifications to the Level 1 or associated criteria). Using the updated logical translation of the Brighton guidelines, we achieved the intended results. In the row ‘Ontology without negation’, we have 223 cases for ‘Level 2 updated’ and 8 results for ‘Level 3 updated’. By comparison the original algorithm misses cases and detects only 178 cases for ‘Level 2 updated’ and 4 results for ‘Level 3 updated’ (20 and 50% missed respectively). Finally, a rather large difference was observed for the category ‘Insufficient evidence’. Running the ABC tool as shown in [19], Botsis et al. found that 488 cases were classified as ‘Insufficient evidence’. However, according to the Brighton guideline, the full label for this category is ‘Reported anaphylaxis with insufficient evidence’, and is meant to identify cases for which there may have been misdiagnosis from the reporting physician, or not enough evidence according to the Brighton criteria to establish the anaphylaxis diagnosis. In the original dataset, only 12 reports were annotated with an ‘anaphylaxis’ MedDRA term (including anaphylaxis-like terms, e.g., anaphylactic reaction, anaphylactic shock,…). Out of those 12 reports, only 3 were lacking supporting evidence as shown in Table 1, column ‘Insufficient evidence’, rows 2 and 3. This results from the fact that the online ABC tool that was used for classification, provides a ‘diagnosis confirmation’ tool, which implies that the user wants to confirm an anaphylaxis diagnosis that they established. Consequently, those 488 cases were incorrectly categorized as ‘reported anaphylaxis with insufficient evidence’.

### Automated Case Screening

**Figure 3:**
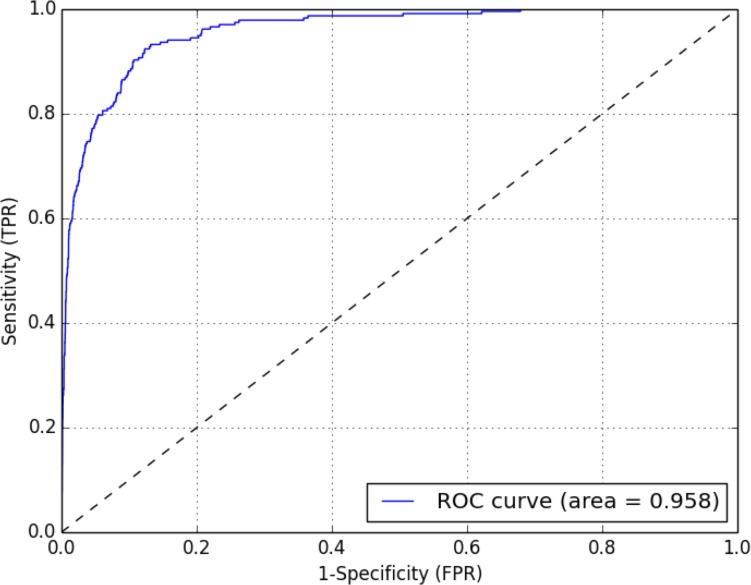
Cosine similarity Receiver Operating Characteristic (ROC) curve. ROC curve showing the sensitivity (True Positive Rate, TPR) vs. 1- Specificity (False Positive Rate, FPR) when measuring cosine similarity of the expanded SMQ built from the existing SMQ and augmented with the terms identified as being significantly correlated with the outcome based on contingency tables. Statistics were computed using the R pROC package [41].

In the previous section we translated the Brighton guidelines into their logical representation, and applied the AERO to automate classification of vaccine adverse event reports from VAERS. As shown in Table 2, while the resulting specificity is very high (97%), the corresponding sensitivity is fairly low (57%). This can however be easily understood if we remember that the Brighton guidelines were never meant for screening, but instead are reporting and diagnosis confirmation guidelines. The guidelines themselves were designed to identify only portion of the cases (low sensitivity) but do so extremely accurately (high specificity). Sensitivity needs to be significantly increased for the purpose of automated identification of rare adverse events. To address the issue of detecting similarity between the diagnosis text and the adverse event reports, we used the well-established information retrieval technique of cosine similarity [26]. Each document (diagnosis query or report) was decomposed into its corresponding vector of terms (e.g., ‘skin rash’, ‘generalized pruritus’). The angle those vectors form can be used to measure the similarity between them: the cosine of the angle is 1.0 for identical vectors and 0.0 for orthogonal ones. Terms in the vectors were weighted using the term frequency-inverse document frequency (tf-idf) scheme, which numerically translates the importance of each term in function of its frequency (tf) in a given document and its frequency in the global dataset (idf). In [19], the authors divide the whole dataset into training and testing subsets, and use the training subset to identify which terms are correlated with the outcome, which they then use to classify reports in the testing set. Rather than creating a bag of words *de novo* based on keyword extraction from a training set of reports, we rather chose to expand on a known, already widely implemented, screening method, i.e., the SMQs. While using the existing anaphylaxis SMQ on its own did not yield satisfactory results in terms of sensitivity, the use of an expanded SMQ provided results that improved upon those described in [19]. To identify which terms statistically correlate significantly with the outcome, we extracted the 2273 different MedDRA terms, and, using the classified dataset, for each built a contingency table and computed the associated *χ* ^2^ and p-value. An *α* level of significance at 0.05 and at one degree of freedom corresponds to a *χ* ^2^ value of 3.841. The 120 MedDRA terms above this threshold were selected (see Table S1), to which we added the 77 terms from the existing SMQ, and then removed duplicates. The remaining 168 MedDRA terms were used to perform a cosine similarity based classification. As shown on Figure 3, this expanded SMQ significantly improves sensitivity (92% against 85% in [19]) with slight increase in terms of specificity (88% against 86%). Our Area Under the Curve (AUC) was also high (0.96) compared to 0.80 in Botsis *et al.*’s training set: using our approach the classifier correctly discriminates between a positive and negative outcome in almost 96% of the cases. Similar results were obtained using a 50/50 training/testing data split: 92% sensitivity (86–96% at 95% CI) and 81% specificity (80–82% at 95% CI) in the testing set, AUC 0.93 (0.9–0.95 at 95% CI). Full classification results are shown in Table 2.

**Table 2:** Comparison of different classification methods.

	Sensitivity (95% CI)	Specificity (95% CI)	AUC (95% CI)
**Brighton Collaboration**			
ABC tool*	0.64 (0.52–0.75)	0.97 (0.96–0.98)	NA
Ontology Classification	0.57 (0.51–0.64)	0.97 (0.96–0.97)	0.77 (0.74–0.80)
IR approach*	0.86 (0.75–0.93)	0.7861 (0.76–0.80)	NA
**SMQ**			
SMQ categories (combined)*	0.54 (0.42–0.66)	0.97 (0.96–0.98)	NA
IR approach*	0.85 (0.73–0.92)	0.86 (0.84–0.87)	NA
Expanded SMQ	0.92 (0.89–0.95)	0.88 (0.87–0.89)	0.96 (0.95–0.97)

*indicates that the result was taken from [19] (values for the testing set). In the Brighton Collaboration section, the ABC tool and ontology-based classification have similar outputs (the small difference in terms of sensitivity can be explained as Botsis et al. split their dataset into training and testing). In the SMQ section, the expanded SMQ yields better results in terms of sensitivity and specificity compared to the existing SMQ categories and the IR approach proposed in [19]. CI: confidence interval.

## Discussion

Our results indicate that using a logical formalization of existing guidelines helps identify missing elements in the reporting pipeline, as well as errors in the interpretation and application of the guidelines. We also found that Brighton guidelines are not optimally suited for case identification in the currently existing reporting systems. Despite having an efficient, standardized and accurate ontological representation of the information, the guidelines were not designed for this purpose. By providing a suitable formalism and method, and encoding multiple versions of the Brighton guidelines, we demonstrated that the AERO can represent multiple guidelines, and allows for immediate comparison of classification across them. Additionally, our work suggests that relying only on the MedDRA encoded anaphylaxis (and associated synonyms such as ‘anaphylactic reaction’) in VAERS [27] may cause severe underestimation of the number of actual cases, as we found that only 12 reports were reported as anaphylaxis in a dataset in which careful manual review identified 236 potentially positive cases. Finally, we demonstrated that automated adverse event screening can reach a very high sensitivity and specificity by building a specific bag of words (SMQ or guideline based), on the best query terms we identified.

### Using an OWL-based Approach

Current state of the art for automated use of the Brighton case definitions is the ABC tool; however as shown above it is not suitable for automated classification. Our approach not only addresses the limitations of the ABC tool, but also provides an open and extensible foundation which can be incorporated into future classification tools. Despite the Brighton guidelines not being optimally suited for the screening problem in the current context, there are multiple benefits in choosing to adopt a logical formalization of the surveillance guidelines considered, and we detail those below. Regarding the choice of the formalism, OWL is an accepted standard for knowledge representation, and comes with a large suite of tools allowing editing, storage and more importantly reasoning is supported by various softwares [21]–[23], [28]–[30]. We demonstrated that even complex guidelines, such as the Brighton Anaphylaxis one, can be encoded using OWL2, and successfully lead to the desired inferences.

#### Limitations of our results

The main limitation of our results is the analysis of the report annotations. The ability to use Natural language Processing (NLP) methods on the textual part would potentially allow further discrimination, and provide supporting evidence in decision making. Additionally, we relied on a mapping between MedDRA and Brighton for part of our classification pipeline. This mapping is subjective and may not be identical to the one another group would produce. Finally, while we could have worked towards increasing the sensitivity/specificity of the classification results using the AERO, we decided that this would change the purpose of the Brighton guidelines and was not desired. However, one could imagine that a ‘Brighton screening guideline’ could be created for that purpose.

#### Formalization of the case definition

Having a formal representation of the guideline, which could be distributed alongside a manuscript, would help both prevent misinterpretation (such as those we observed as a result of not taking into consideration the underlying assumption that it performs diagnosis confirmation), and enable homogenized implementation in electronic systems of the chosen standard. Several studies [31], [32] rely on the number of adverse events detected in VAERS to hypothesize whether their rate is higher than expected with a certain vaccine. It is not currently possible to compare those studies, not even in cases in which they concern the same adverse event. For example, in [33], the authors define anaphylaxis in a less restrictive way than the Brighton criteria. In [34], yet another set of criteria is used, even though the two papers share authors. In [35], the authors acknowledge that different criteria were used for anaphylaxis identification, including the Brighton criteria, but conclude that they could not use the latter as this was not compatible with existing published reports. It is critical to ensure that not only reporters use standard for reporting, but also that medical officer know which standards were used, and be able to compare different ones. This is not only crucial for VAERS, but more importantly critical to reach the goal of having an international assessment of vaccine safety [36]. Finally, several projects have been recently concerned with addressing the need for reporting guidelines, such as the CARE guidelines [37], the PROSPER Consortium guidance document [38] or the integration of guidelines into asthma electronic record [39], the latter two specifically advocating for the use of taxonomies.

#### Use of the ontology for reporting

Another way to improve detection of adverse events is to standardize the reporting step. Currently, reports are centralized and then annotated with MedDRA terms by specialized coders. These individuals do not see the patient, and if deemed it necessary, they need to request more detailed medical reports after the fact. If we were able to provide to the person reporting the event, at data entry time, a tool that allows unambiguous and consistent reporting of the signs and symptoms they observe, we would be able to capture this information with the submitted report, and would subsequently not need to devise complex data-mining of the reports to classify them. Using the ontology at data entry time would provide two distinct advantages: (1) the ontology provides textual definition for all the criteria terms and (2) the ontology can be used to enforce consistency checking at data entry time. Regarding (1), one of the requisite of our collaboration with the Public Health Agency of Canada (PHAC) was that the resource developed would be usable by human as well as machines. We therefore not only encoded the logical axioms derived from the Brighton case definitions, but also added the human readable labels and textual definitions, most of those provided from [13]. Regarding (2), upon development of a data capture form capturing the Brighton criteria, the ontology can be used locally to check whether conditions for the diagnosis establishment are met. For example, when a physician reports ‘anaphylaxis’, the system could automatically ask for relevant signs and symptoms and store whether they have been observed or not. This would also help with respect to capturing whether the information that is not present in the report is missing or unknown.

#### Going forward: proposed implementation

As we are considering rare adverse events, we need to ensure all possibly potential cases are retrieved, and to the best of our knowledge our results are the best obtained to date. We recommend a hybrid approach where both the SMQ information retrieval method and the AERO classification approach be used in parallel. The output of the high sensitivity classifier allows for extraction of a subset of the original dataset, even though we know there will be false positives (12.3%). In our case, we were able to rightfully discard 5082 true negatives. If intersecting, the Brighton confirmed cases can be subtracted from this, allowing curators to focus on the remaining reports. Also, a fast screening method when data is being sent in would allow to automatically identify potentially positive cases, at which point a more detailed form (such as the Brighton-based reporting form from PHAC) can be immediately provided to the reporter.

## Conclusion

By standardizing and improving the reporting process, we were able to automate diagnosis confirmation. By allowing medical experts to prioritize reports such a system can accelerate the identification of adverse reactions to vaccines and the response of regulatory agencies. Future reporting systems should provide a web-based interface (or a form in their electronic data capture systems) that reflects the criteria being used for case classification. This would help ensure that the information being captured is standardized and that potentially missing information can be immediately added by adding consistency checking tests. While this manuscript provides way of improving standardization in passive, spontaneous reporting systems such as VAERS, other avenues can be explored to improve surveillance, such as promoting active systems [40]. At a minimum, providers of guidelines should recognize issues such as those we describe, and commit to provide logical representations of their work. Based on our partnership and results, the Brighton Collaboration is moving towards providing such a representation for their case definitions.

## Availability

The AERO project, including documentation, links to the ontology and code is available at http://purl.obolibrary.org/obo/aero. AERO can be imported into any OWL ontology using the Ontology IRI http://purl.obolibrary.org/obo/aero.owl. The ontology is listed in the OBO library at http://obofoundry.org/cgi-bin/detail.cgi?id=AERO and in the BioPortal at http://bioportal.bioontology.org/ontologies/1580.

## Supporting Information

Table S1 MedDRA terms with a chi-square value over 3.841.
(PDF)
