# Supplementary material for: The Logic of Surveillance Guidelines: An Analysis of Vaccine Adverse Event Reports from an Ontological Perspective
Source: PLoS One. 2014 Mar 25;9(3):e92632. doi: 10.1371/journal.pone.0092632 (PMC3965435; doi:10.1371/journal.pone.0092632)
Supplement: Table S1 — MedDRA terms with a chi-square value over 3.841. (PDF) [file pone.0092632.s001.pdf]

Table S1: MedDRA terms with a chi-square value over 3.841

MedDRA term	Chi-square	P-value
Hypersensitivity	1578.605353	0
Dyspnoea	553.3557	2.34E-122
Throat tightness	551.5865009	5.69E-122
Pruritus	297.906177	9.42E-67
Chest discomfort	296.2635345	2.15E-66
Pharyngeal oedema	251.7630256	1.07E-56
Urticaria	231.0682725	3.49E-52
Wheezing	205.1667372	1.56E-46
Swelling face	203.0038003	4.62E-46
Anaphylactic reaction	198.3924991	4.68E-45
Oedema	181.4914781	2.29E-41
Swelling	179.028501	7.90E-41
Lip swelling	177.3909311	1.80E-40
Discomfort	160.1597406	1.04E-36
Swollen tongue	157.5517954	3.88E-36
Throat irritation	154.4938506	1.81E-35
Eye swelling	141.3551256	1.35E-32
Tic	122.0267653	2.28E-28
Dysphagia	83.93452989	5.11E-20
Vaccination complication	81.70570956	1.58E-19
Rash	68.93363732	1.02E-16
Anxiety	56.33309817	6.12E-14
Paraesthesia oral	51.40599746	7.51E-13
Dermatitis allergic	50.13558624	1.43E-12
Oxygen saturation	49.73241883	1.76E-12
Flushing	49.3121747	2.18E-12
Allergy to vaccine	44.76216274	2.22E-11
Heart rate increased	41.07021225	1.47E-10
Electrocardiogram normal	40.11780423	2.39E-10
Palpitations	37.25210863	1.04E-09
Dysphonia	36.7245365	1.36E-09
Erythema	34.31261596	4.69E-09
Oxygen saturation normal	33.65197646	6.59E-09
Cough	33.12717418	8.63E-09
Electrocardiogram	32.54342042	1.17E-08
Chest pain	31.8973366	1.63E-08
Eye pruritus	31.06355091	2.50E-08
Oedema peripheral	28.64424038	8.70E-08
Heart rate	28.6141026	8.83E-08

MedDRA term	Chi-square	P-value
Oral pruritus	28.13879224	1.13E-07
Idiopathic urticaria	26.77190018	2.29E-07
Angioedema	24.88169145	6.10E-07
Tachycardia	24.1470991	8.93E-07
Ocular hyperaemia	23.56285888	1.21E-06
Dizziness	21.90501031	2.86E-06
Pruritus generalised	20.41537534	6.23E-06
Hyperventilation	20.28914823	6.66E-06
X-ray normal	18.62066883	1.59E-05
Rash erythematous	17.79906026	2.46E-05
Chest X-ray normal	17.02743339	3.68E-05
Non-cardiac chest pain	16.87767466	3.99E-05
Oxygen saturation decreased	16.50541696	4.85E-05
Adverse drug reaction	15.84086837	6.89E-05
Asthma	14.94011526	0.000110978
Hypertension	13.76604066	0.000207045
Rhinitis	13.68760651	0.000215874
Food allergy	13.58133526	0.000228446
Rash macular	12.90979478	0.000326867
Blood glucose increased	12.39650931	0.000430137
Bronchial hyperreactivity	11.95078269	0.000546244
Oedema mouth	11.95078269	0.000546244
Dry throat	11.78175253	0.000598141
Respiratory rate	11.513761	0.000690829
Chest X-ray	10.74988156	0.00104286
Paraesthesia	10.46235549	0.001218318
Tension	9.777425891	0.001766675
Pyrexia	9.460175584	0.00209981
Feeling abnormal	9.424379867	0.002141195
Presyncope	9.414183846	0.002153134
Altered state of consciousness	9.010832195	0.002683842
Respiratory rate decreased	9.010832195	0.002683842
Rhinitis allergic	9.010832195	0.002683842
Red blood cell count normal	9.010832195	0.002683842
Respiration abnormal	9.010832195	0.002683842
Skin test	9.010832195	0.002683842
X-ray	8.958551384	0.002761738
Eyelid oedema	8.395515718	0.003761478
Hypoaesthesia oral	8.260899726	0.004050805
Feeling hot	8.222546332	0.004137311
Face oedema	8.081313552	0.004472402
Immediate post-injection reaction	7.72106081	0.005458032

MedDRA term	Chi-square	P-value
Blood glucose	7.72106081	0.005458032
Stridor	7.064359153	0.007863244
No reaction on previous exposure to drug	6.745371787	0.009399119
Blood pressure	5.971226848	0.014541161
Dermatitis	5.813875639	0.015900215
Feeling jittery	5.685593271	0.017104755
Lymph node palpable	5.624025895	0.017715909
Activated partial thromboplastin time shortened	5.624025895	0.017715909
Panic disorder	5.624025895	0.017715909
Skin test negative	5.624025895	0.017715909
Arrhythmia supraventricular	5.624025895	0.017715909
Steroid therapy	5.624025895	0.017715909
Oropharyngeal spasm	5.624025895	0.017715909
Soft tissue inflammation	5.624025895	0.017715909
Laryngospasm	5.624025895	0.017715909
Vaccination site erythema	5.624025895	0.017715909
Barium swallow normal	5.624025895	0.017715909
Lip discolouration	5.624025895	0.017715909
Plantar fasciitis	5.624025895	0.017715909
Food aversion	5.624025895	0.017715909
Computerised tomogram thorax normal	5.624025895	0.017715909
Oropharyngeal swelling	5.624025895	0.017715909
Vaccination site pruritus	5.624025895	0.017715909
Scan myocardial perfusion normal	5.624025895	0.017715909
Vasoconstriction	5.624025895	0.017715909
Blood electrolytes decreased	5.624025895	0.017715909
Venous thrombosis	5.624025895	0.017715909
Troponin	5.474670434	0.019293998
Pain in extremity	5.123595971	0.023602658
Bronchitis	4.782141775	0.028756334
Myalgia	4.763685188	0.029066252
Blood pressure decreased	4.744564425	0.029390993
Metabolic function test	4.672009897	0.030658028
Oxygen supplementation	4.300705957	0.038096556
Productive cough	4.18625334	0.040753069
Serum sickness	3.874258039	0.049031977
Hypokalaemia	3.874258039	0.049031977
Bronchospasm	3.874258039	0.049031977
Hypoventilation	3.874258039	0.049031977
